# Effect of Children’s Autism Spectrum Disorder Severity on Family Strain and Sleep Quality: A Cross-Sectional Online Survey in the U.S.

**DOI:** 10.1007/s10803-022-05457-7

**Published:** 2022-02-03

**Authors:** Gonzalo Durán-Pacheco, Mariabeth Silkey, Michelle Johnson, Chuang Liu, Susanne Clinch, Kiely Law, Georg Loss

**Affiliations:** 1 F. Hoffmann-La Roche Ltd., Basel, Switzerland; 2 Roche Products Ltd., Hexagon Place, 6 Falcon Way, Shire Park, Welwyn Garden City, AL7 1TW UK; 3 Kennedy Krieger Institute, Baltimore, MD USA; 4 Johns Hopkins University School of Medicine, Baltimore, MD USA

**Keywords:** Autism spectrum disorder, Child health, Autism Impact Measure, Caregiver strain, Sleep quality, Severity

## Abstract

To better understand the impact of children’s autism spectrum disorder (ASD) severity on families, we evaluated pathways through which ASD severity affected child sleep quality, caregiver strain, and caregiver sleep quality. In a cross-sectional analysis through the U.S.-wide Simons Foundation Powering Autism Research for Knowledge (SPARK) cohort. Participants were caregivers of dependents with ASD aged 3–17 years (N = 3150). We found that increased severity strongly affects caregiver strain and child sleep quality. Child sleep quality was a minor mediator of increasing caregiver strain. Caregiver sleep quality depended on ASD severity only through child sleep quality and caregiver strain. Interventions aimed at improving child sleep quality or reducing caregiver strain could positively impact families of children with ASD.

**Supplementary Information:**

The online version contains supplementary material available at 10.1007/s10803-022-05457-7.

Autism spectrum disorder (ASD) is a neurodevelopmental condition that affects the lives of individuals and, as a result, impacts the lives of their caregivers and families. Since the onset of ASD generally occurs in childhood, caregivers play a key role in seeking diagnosis, treatment, and social support (Brannan et al., 2012). Caregivers have the greatest responsibility for the everyday care of the child with ASD, and are often relied upon to help implement therapy for the child (Bloch & Weinstein, 2009). The experiences of families of children with ASD are varied (Bloch & Weinstein, 2009; Kirby et al., 2019) but generally these are more stressful than those of neurotypical children (Hayes & Watson, 2013; Karst & Van Hecke, 2012; Yang et al., 2021). Many factors can influence reactions to caregiving challenges such as the child’s age, timing of diagnosis, or social support (Bekhet et al., 2012; Iadarola et al., 2018). The extra responsibilities needed to optimally care for the child with ASD can be difficult for families and can impact family functioning (i.e., the way that families manage daily life, extent of effective communication, and cultivate positive relationships), cause loss of personal time, less participation in recreational activities, worry, and fatigue, among other stresses (Bloch & Weinstein, 2009; Brannan et al., 1997, 2012; Estes et al., 2013; Jellett et al., 2015). These, in turn, have negative consequences for caregiver mental health and quality of life (Khanna et al., 2011; Yorke et al., 2018). The significant costs associated with raising a child with ASD include indirect costs such as loss or disruption of work for parents and caregivers (Buescher et al., 2014), which can also place additional stress on families.

Caregiver strain, also referred to as ‘parental burden’, is the additional demands and responsibilities on caregivers and the perceived impact of caregiving responsibilities on the mental health, physical health, relationships, and finances of the family (Brannan et al., 1997, 2012; Khanna et al., 2012; Sales, 2003). Among caregivers of children with ASD, caregiver strain is persistent over long periods of time (Lindly et al., 2021), has been associated with lower mental health-related quality of life (Khanna et al., 2011), and this association has been shown to be mediated by maladaptive coping skills of the caregivers (Lee et al., 2019).

Furthermore, sleep disorders occur frequently in children with ASD, with studies reporting prevalence rates of 64–93% (Carmassi et al., 2019; Richdale & Schreck, 2009). Studies have shown that increased ASD severity raises the likelihood of sleep problems and can worsen the severity of sleep problems (Adams et al., 2014; Carmassi et al., 2019; Tudor et al., 2012). Parents of children with developmental disorders report worse sleep quality compared with parents of typically developing children and parental stress is a predictor of poor sleep quality (Gallagher et al., 2010). For parents of children with ASD, maternal mental health and stress has been strongly associated with sleep quality (Lee, 2013). Poor child sleep quality also impacts parental stress and mental health (Abdullah et al., 2021), and treatment of insomnia in children with ASD has been shown to improve some aspects of parental stress (Malow et al., 2012).

Caregiver strain and parental stress not only impacts caregivers but can also have a negative impact on the parent–child relationship, which in turn can impede child development (Crowell et al., 2019). Elevated parent stress and ASD symptoms can also have a bidirectional escalating effect whereby parents who are stressed by their children’s ASD symptoms alter their parenting style in a way that leads to an increase in the severity of ASD symptoms and causes even more parental stress (Rodriguez et al., 2019; Yorke et al., 2018). Of particular concern are results from longitudinal studies of parental stress (Rodriguez et al., 2019) and caregiver strain (Lindly et al., 2021) among caregivers of children with ASD, demonstrating that high stress is, on average, stable across several years. It is still unknown how the child’s ASD severity translates into downstream effects within the family, i.e., by modifying child sleep quality first, in turn affecting caregiver sleep, or perhaps by increasing caregiver stress first, and only then reducing sleep quality of caregivers. In order to better understand the impact of ASD on the family, specifically the caregivers, we investigated the pathways through which the child’s overall ASD severity (impact and frequency of core symptoms, ASD-related major adversities, and caregiver-reported severity) affects caregiver strain, child sleep quality, and caregiver sleep quality. A better understanding of the pathways impacted by the child’s ASD severity could lead to identifying areas where interventions are most needed, and ultimately have a positive impact on families and caregivers of children with ASD.

## Methods

### Study Design and Participants

This was a cross-sectional analysis of electronic caregiver surveys, sampled from the U.S.-wide Simons Foundation Powering Autism Research for Knowledge (SPARK) online research initiative (SPARK Consortium, 2018). Participant recruitment, online consent, and data collection were facilitated by SPARK. A baseline study was conducted in 2017 with the same population as this study; details of recruitment and data collection were published previously (Houghton et al., 2019; Monz et al., 2019). Eligibility criteria for this study were as follows: caregivers of individuals with ASD aged 3–17 years, who participated in the baseline study, and submitted the Autism Impact Measure (AIM) (Kanne et al., 2014) questionnaire with ≤ 20% of missing items at baseline. Eligible participants were sent email invitations and participants consented online. The research protocol was approved by Western Institutional Review Board (tracking number 20181254) and the study complied with the Guidelines for Good Pharmacoepidemiology (International Society for Pharmacoepidemiology, 2015).

The following online questionnaires were completed by participants between September 12 and October 16, 2018: AIM, Caregiver-reported Global Impression of Severity (CaGI-S), and the Caregiver Strain Questionnaire-Short Form 7 (CGSQ-SF7), as well as additional information on sleep quality and ASD-related major adversities. Demographics, socioeconomic, and geographic data (as in Table 1) were recorded during the baseline survey in 2017 and 2018. Data at baseline were used to describe the study population and control for confounding.

**Table 1:** Child, caregiver, and household characteristics stratified by AIM score quartile

Characteristic	1st Quartile	2nd Quartile	3rd Quartile	4th Quartile	Overall
*N*	801	783	781	785	3,150
*Child with ASD*					
Male, *n* (%)	647 (81.2)	624 (80.3)	606 (78.4)	630 (80.7)	2,507 (80.1)
Age, median years [IQR]	10.0 [7.0–14.0]	10.0 [7.0–13.0]	9.0 [6.0–12.0]	8.5 [6.0–12.0]	9.0 [7.0–13.0]
*Race/ethnicity group*, *n* (%)					
Non-White/Hispanic	37 (4.6)	39 (5.0)	39 (5.0)	48 (6.1)	163 (5.2)
Non-White/non-Hispanic	126 (15.7)	117 (14.9)	118 (15.1)	146 (18.6)	507 (16.1)
White/Hispanic	91 (11.4)	73 (9.3)	70 (9.0)	81 (10.3)	315 (10.0)
White/non-Hispanic	547 (68.3)	554 (70.8)	554 (70.9)	510 (65.0)	2,165 (68.7)
Time since ASD diagnosis, median years [IQR]	5.0 [3.0–8.0]	4.0 [3.0–7.0]	4.0 [3.0–7.0]	5.0 [3.0–8.0]	5.0 [3.0–8.0]
Child takes drug (any) for their ASD, *n* (%)	217 (27.5)	254 (32.9)	311 (40.1)	312 (40.3)	1,094 (35.2)
*Caregiver*					
Male, *n* (%)	39 (4.9)	37 (4.7)	48 (6.2)	51 (6.5)	175 (5.6)
Caregiver age, median years [IQR]	41.0 [36.0–45.0]	39.0 [35.0–44.0]	39.0 [34.0–44.0]	38.0 [34.0–43.0]	39.0 [34.0–44.0]
*Employment status*, *n* (%)					
Unemployed	19 (2.4)	16 (2.0)	29 (3.7)	25 (3.2)	89 (2.8)
Student	13 (1.6)	20 (2.6)	16 (2.1)	26 (3.3)	75 (2.4)
Part-time	156 (19.5)	149 (19.1)	150 (19.3)	133 (17.0)	588 (18.7)
Full-time	381 (47.7)	346 (44.2)	329 (42.3)	304 (38.9)	1,360 (43.3)
Retired	4 (0.5)	6 (0.8)	4 (0.5)	9 (1.2)	23 (0.7)
Homemaker	208 (26.1)	224 (28.6)	233 (30.0)	259 (33.2)	924 (29.4)
Other	17 (2.1)	21 (2.7)	16 (2.1)	25 (3.2)	79 (2.5)
Caregiver has a partner, *n* (%)	650 (81.2)	639 (82.2)	615 (79.3)	601 (77.3)	2,505 (80.0)
*Household*					
*Household income in USD*, *n* (%)					
< $50,000	219 (27.3)	261 (33.3)	317 (40.6)	340 (43.4)	1,137 (36.1)
$50,000–$99,999	260 (32.5)	292 (37.3)	244 (31.2)	238 (30.3)	1,034 (32.8)
≥ $100,000	283 (35.3)	200 (25.5)	193 (24.7)	180 (22.9)	856 (27.2)
Prefer not to answer	39 (4.9)	30 (3.8)	27 (3.5)	26 (3.3)	122 (3.9)
*Number in household, including caregiver and child with ASD*, *n* (%)					
2–3	238 (29.8)	201 (25.7)	229 (29.4)	230 (29.3)	898 (28.6)
4	316 (39.5)	339 (43.4)	288 (36.9)	290 (37.0)	1,233 (39.2)
≥ 5	246 (30.8)	241 (30.9)	263 (33.7)	264 (33.7)	1,014 (32.2)
*U.S. region*, *n* (%)					
West	188 (23.5)	202 (25.8)	184 (23.6)	206 (26.3)	780 (24.8)
South	292 (36.5)	282 (36.1)	295 (37.8)	281 (35.8)	1,150 (36.6)
Midwest	167 (20.9)	192 (24.6)	178 (22.8)	166 (21.2)	703 (22.3)
Northeast	153 (19.1)	106 (13.6)	123 (15.8)	131 (16.7)	513 (16.3)
*Metropolitan service area*, *n* (%)					
Metropolitan	655 (81.8)	650 (83.0)	618 (79.1)	633 (80.6)	2,556 (81.1)
Micropolitan	87 (10.9)	85 (10.9)	105 (13.4)	92 (11.7)	369 (11.7)
Unknown	59 (7.4)	48 (6.1)	58 (7.4)	60 (7.6)	225 (7.1)

Score ranges of the AIM distribution quartiles: 1st: (83–168); 2nd: (168–206); 3rd: (206–244); 4th: (244–410). Higher AIM scores indicate increased ASD symptom impact and frequency. Demographic variables drawn from the 2017 baseline survey

*AIM* Autism Impact Measure, *ASD* autism spectrum disorder, *IQR* interquartile range, *USD* United States Dollars

### Autism Impact Measure (AIM)

AIM is a caregiver-reported questionnaire designed to measure subtle changes in core ASD symptoms in individuals aged 3–18 years old (Kanne et al., 2014; Mazurek et al., 2018). The AIM consists of 41 items that are each rated for both frequency and impact on a 5-point Likert-type scale. Caregivers rate how often their child exhibited a behavior in the previous 2 weeks ranging from 1 (“Never”) to 5 (“Always”) for the frequency scale and how much each of the behaviors interfered with their child’s everyday functioning from 1 (“Not at all”) to 5 (“Severely”) for the impact scale. The range of possible scores for the total AIM is 82–410 (Kanne et al., 2014; Mazurek et al., 2018). Since the total AIM score combines both frequency and impact of ASD core symptoms, and higher scores indicate greater impact of ASD symptoms (Houghton et al., 2019; Mazurek et al., 2018), it was used as a proxy for ASD symptom severity.

### Caregiver-Reported Global Impression of Severity (CaGI-S)

Overall ASD severity was assessed by the caregiver using a CaGI-S Autism scale, an adaptation of the Clinician Global Impressions Scale for Severity (Busner & Targum, 2007), consisting of items rated on a 5-point scale for behaviors observed during the previous 2 weeks. The single item score relating to the caregiver’s impression of the child’s overall severity was used; it was scored from 1 (“Not at all severe”) to 5 (“Extremely severe”).

### ASD-Related Major Adversities

Health- or ASD-related adversities that occurred during the previous 12 months (presence versus absence) were reported by the caregiver, including whether the child was verbal, child experienced other mental health or psychiatric problems, child hospitalized for mental health problems, child visited emergency room (ER) for mental health problems, child eloped, and child suspended/expelled from school. The above listed events were considered ASD-related major adversities in the statistical analyses.

### Caregiver Strain Questionnaire-Short Form 7 (CGSQ-SF7)

Caregiver strain was measured with the CGSQ-SF7, a seven-item version (Bickman et al., 2010; Brannan et al., 2012) of the full 21-item questionnaire designed for caregivers and parents to self-report on their perceived burden associated with caring for a child with emotional or behavioral disorders (Brannan et al., 1997; Khanna et al., 2012). The CGSQ (Brannan et al., 1997) is a reliable and valid instrument to assess strain among caregivers of children with ASD (Iadarola et al., 2018; Khanna et al., 2011, 2012; Lee et al., 2019). The CGSQ-SF7 assesses objective strain and subjective internalized strain and demonstrated comparable reliability and validity to the original questionnaire (Brannan et al., 2012). Caregivers rate “How much of a problem were the following?” for each item in the previous month on a 5-point scale ranging from 1 (“Not at all”) to 5 (“Very much”); the items include interruption of personal time, missing work or neglecting duties, financial strain for the family, disruption of family relationships, how sad or unhappy the caregiver feels, how worried the caregiver is for the child’s future, and how tired or strained the caregiver feels. Total strain scores were derived by summing the score of each item, resulting in a total score ranging from 7 to 35.

### Sleep Quality

Child and caregiver sleep quality over the previous 2 weeks was rated separately by the caregiver on a single-item question for each using a 10-point scale from 0 (“Best imaginable sleep”) to 10 (“Worst imaginable sleep”). These variables were scaled as follows: sleep score = (value – min) / (max – min) bounded between 0 and 1. Resulting scores were standardized to a mean 0 and standard deviation 1.

### Data Analysis

Descriptive analyses were carried out to gain an understanding of the study population and data quality. Categorical variables were described using counts and proportions. Continuous variables were described using median and interquartile range (IQR). Fisher’s exact and Kruskal–Wallis tests were conducted as univariate statistical tests to compare categorical and continuous variables respectively across groups of interest. Multiple correspondence analysis (Lebart, 1995) was applied to explore and display relationships among survey responses of categorical variables regarding ASD-related major adversities, caregiver’s assessment of overall severity, and caregiver strain.

**Fig. 1:**
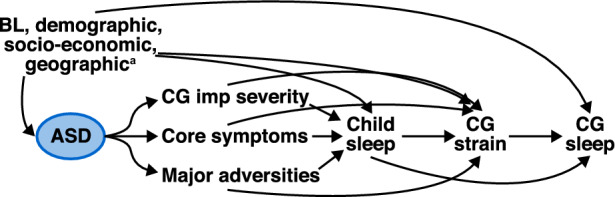
Directed acyclic graph with the hypothesized relationships between ASD core symptoms, ASD-related major adversities, and ASD overall caregiver impression of severity, with child sleep quality, caregiver strain, and caregiver sleep quality. ^a^Baseline demographics, socioeconomic, and geographic characteristics for household, caregiver, and child. *ASD* autism spectrum disorder, *BL* baseline, *CG* caregiver, *imp* impression

We postulated a model describing how ASD relates to sleep quality of the child, sleep quality of the caregiver, and to caregiver strain as shown in Fig. 1 (alternative competing models are shown in Supplementary Information Figs. S1a–b). We evaluated ASD severity in three alternative ways: (1) impact of ASD core symptoms (AIM total scores), (2) caregiver impression of ASD severity (CaGI-S), and (3) ASD-related major adversities (as listed above). Our proposed model implies that, in addition to the impact of individual and family background factors (demographic, geographic, socioeconomic, or others), the level of ASD severity affects child sleep quality and caregiver strain directly. Part of these effects on caregiver strain are mediated by child sleep quality. Additionally, greater ASD severity is linked to a decline in caregiver sleep quality only through effects mediated by child sleep quality and caregiver strain.

The proposed model was expressed as a system of structural equations where response variables were fitted as a function of parental nodes conditioned in all cases on child, caregiver, and household baseline, and demographic characteristics (variables in Table 1). Multivariable linear models were fitted to estimate direct effects. Consistency between the hypothesized models (Fig. 1, Supplementary Information Figs. S1a–b) and our empiric data was assessed by testing the implicit conditional independence assumptions. For instance, in Fig. 1, impact of ASD symptoms, impression of severity, and ASD-related major adversities are each d-separated from caregiver quality of sleep, i.e., independent, after adjusting for baseline and demographics, child sleep quality, and caregiver strain. The fraction of effects mediated (or not) by intermediate variables was obtained using mediation analysis (VanderWeele, 2015). All quantitative variables were standardized to a mean 0 and standard deviation 1, therefore regression coefficients are standardized weights interpreted as changes in 1 standard deviation unit of the response variable per change in 1 standard deviation unit of the explanatory variable. Statistical models were fitted without missing data imputation.

All analyses were conducted in the statistical software R Core Team (2019) version 3.6.3. Mediation analysis was conducted using Markov Chain Monte Carlo methods, Gibbs sampling, as implemented in JAGS, and executed using the R library R2jags. For this analysis, uninformative priors were used for all model parameters (Normal and Inverse Gamma for location and scale parameters respectively). Model convergence was assessed by running three chains, a total of 20,000 iterations after 3,000 burn-in.

## Results

### Study Population

**Fig. 2:**
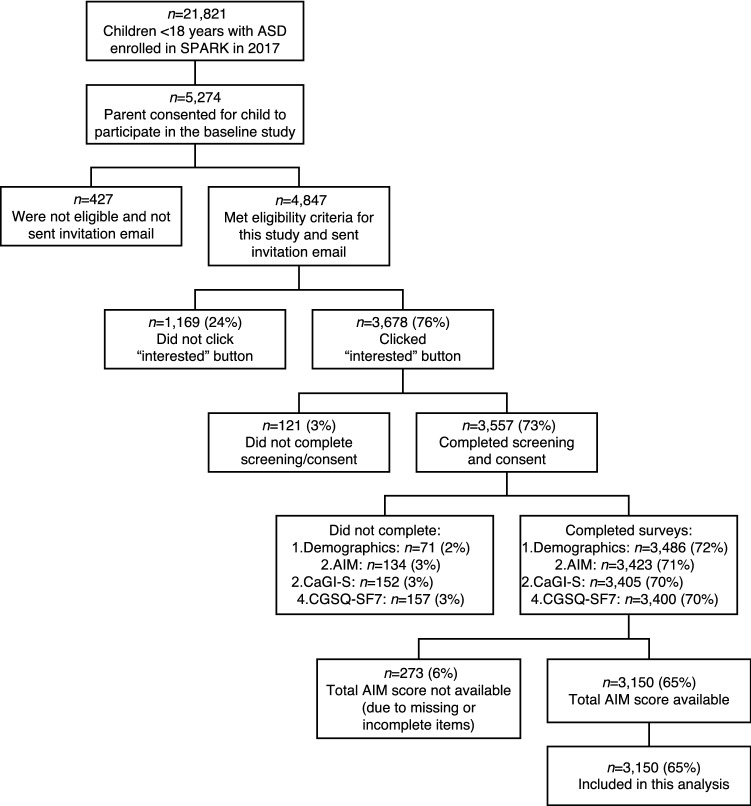
Participant flow diagram. *Note* parentheses show % of total participants sent the invitation email. *AIM* Autism Impact Measure, *ASD* autism spectrum disorder, *CaGI-S* Caregiver-reported Global Impression of Severity Autism survey, *CGSQ-SF7* Caregiver Strain Questionnaire-Short form 7, *SPARK* Simons Foundation Powering Autism Research for Knowledge

Of the 4847 caregivers who met the eligibility criteria and were invited, 3678 (76%) registered interest in participating in this study, and 3557 (73%) completed screening and consent (Fig. 2). The analysis population consisted of 3150 (65%) individuals with ASD under 18 years who had total AIM scores available (no missing items) and completed the CGSQ-SF7 and CaGI-S.

Most participants’ children were male (80%) and of White/non-Hispanic race/ethnicity (68.7%; Table 1). Median age was 9 years (range 3–17 years, IQR 7–13 years) and median time since ASD diagnosis was 5 years (IQR 3–8 years). Overall, 35.2% of children were taking at least one medication prescribed for their ASD symptoms, and the proportion of medication use increased across AIM total score quartiles (i.e., increasing impact of ASD). Caregivers were predominantly parents of the children with ASD (98.7%) and female (94.4%) with a median age of 39 years (IQR 34–44 years). Overall, 43.3% of caregivers were employed full-time; however, this proportion noticeably decreased inversely with higher AIM score quartiles. Most families were from metropolitan areas (81.1%) and all four U.S. regions were represented. The proportion of children from households with incomes ≥ 100,000 USD decreased across the AIM score quartiles, i.e., children with ASD from high-income families had less symptom impact from their ASD (Table 1).

### Distribution of Strain, Severity, and Sleep Quality Scores

Total caregiver strain score (CGSQ-SF7) noticeably increased across AIM score quartiles indicating that caregiver strain correlated with the degree of their child’s impact of ASD symptoms (Table 2). 57.8% of caregivers reported that their child’s ASD was at most “Somewhat severe” (CaGI-S) and caregiver impression of severity was strongly associated with higher AIM score quartiles (*p* < 0.01). Most participant children were verbal (86.6%), 53.4% had other mental health or psychiatric comorbidities, and 23.4% had eloped. Some ASD-related major adversities showed a correlation with the degree of ASD symptoms as measured by the AIM, in particular children who were non-verbal, or had eloped, or those with other mental comorbidities (*p* < 0.01, Table 2).

**Table 2:** Total caregiver strain, caregiver-reported ASD severity, ASD-related major adversities, and sleep quality stratified by AIM score quartile

Characteristic	1st Quartile	2nd Quartile	3rd Quartile	4th Quartile	Overall	*p* value^a^
*N*	801	783	781	785	3,150	
Total caregiver strain (CGSQ-SF7) score^b^ median [IQR]	15 [11–19]	20 [16–24]	22 [18–27]	27 [23–31]	21 [16–27]	< 0.0001
*Caregiver impression of overall ASD severity (CaGI-S), n (%)*						< 0.0001
Not at all severe	463 (58.3)	165 (21.2)	68 (8.7)	22 (2.8)	718 (22.9)	
Somewhat severe	260 (32.7)	381 (49.0)	308 (39.6)	144 (18.4)	1,093 (34.9)	
Moderately severe	67 (8.4)	198 (25.5)	340 (43.7)	377 (48.3)	982 (31.4)	
Very severe	4 (0.5)	27 (3.5)	55 (7.1)	168 (21.5)	254 (8.1)	
Extremely severe	0 (0.0)	6 (0.8)	7 (0.9)	70 (9.0)	83 (2.7)	
Child is verbal, *n* (%)	786 (98.1)	718 (91.7)	683 (87.6)	538 (68.8)	2,725 (86.6)	< 0.0001
Other mental comorbidities present, *n* (%)	374 (47.7)	409 (53.5)	435 (57.6)	413 (55.1)	1,631 (53.4)	0.001
Child eloped, *n* (%)	62 (7.8)	131 (16.8)	209 (26.9)	332 (42.5)	734 (23.4)	< 0.0001
Child hospitalized for mental health care, *n* (%)	9 (1.1)	20 (2.6)	21 (2.7)	21 (2.7)	71 (2.3)	0.068
Child seen in ER for mental health care, *n* (%)	19 (2.4)	26 (3.3)	39 (5.0)	37 (4.7)	121 (3.8)	0.019
Child suspended or expelled from school, *n* (%)	39 (4.9)	39 (5.0)	61 (7.8)	51 (6.5)	190 (6.0)	0.046
Caregiver sleep quality^c^ median [IQR]	4 [3, 6]	5 [3.2, 6]	5 [4, 7]	6 [5, 7]	5 [4, 7]	< 0.0001
Child sleep quality^c^ median [IQR]	3 [2, 5]	4 [3, 6]	5 [3, 6]	5 [4, 7]	4 [3, 6]	< 0.0001

Ranges of the AIM distribution quartiles: 1st: (83–168), 2nd: (168–206), 3rd: (206–244), 4th: (244–410). Higher AIM scores indicate increased ASD symptom impact and frequency

*AIM* Autism Impact Measure, *ASD* autism spectrum disorder, *CaGI-S* Caregiver-reported Global Impression of Severity Autism survey, *CGSQ-SF7* Caregiver Strain Questionnaire-Short form 7, *ER* emergency room, *IQR* interquartile range

^a^*p* values from Fisher’s exact tests and Kruskal–Wallis tests for categorical and numeric variables respectively

^b^Total caregiver strain (CGSQ-SF7) score range 7–35 with higher scores indicating greater strain

^c^Sleep quality scores range from 1 (“Best possible”) to 10 (“Worst possible”)

**Fig. 3:**
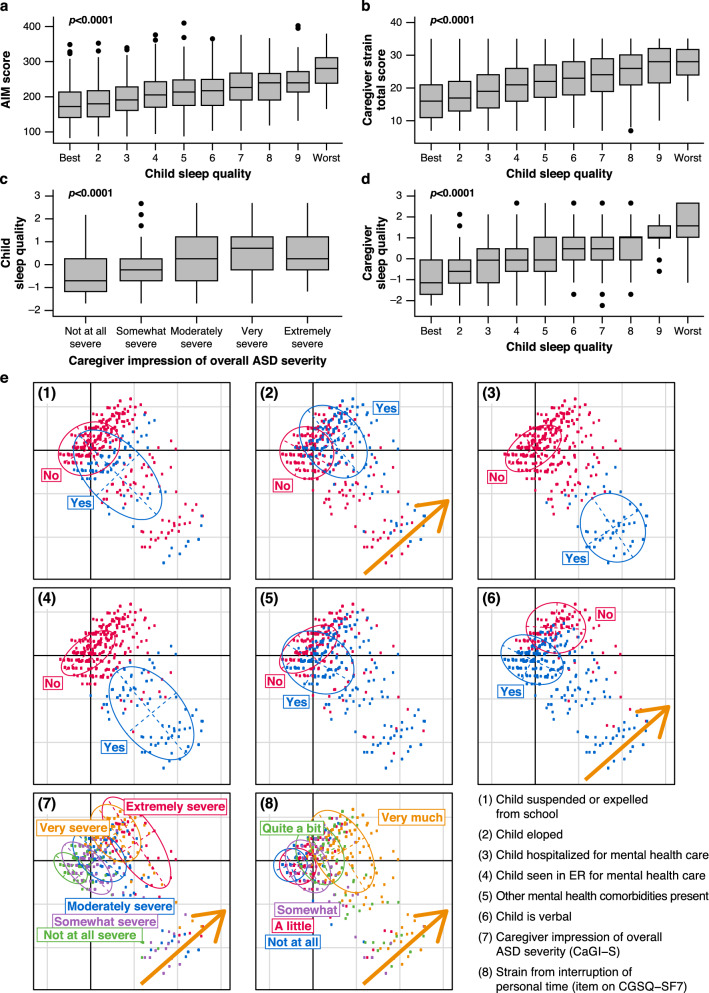
Distribution of AIM total score, child, and caregiver sleep quality, and caregiver-reported ASD severity, ASD-related major adversities, and caregiver strain. **a** Distribution of AIM scores by child sleep quality categories; **b** distribution of caregiver strain (CGSQ-SF7) scores by child sleep quality categories; **c** distribution of child sleep quality standardized scores by caregiver impression of overall ASD severity categories (CaGI-S); **d** distribution of caregiver sleep quality standardized scores by child sleep quality categories; **e** multiple correspondence analysis plot of health and ASD-related major adversities (suspended or expelled from school; eloped or strayed; hospitalized for mental health care; seen in ER for mental health care; other mental comorbidities present; child is verbal), ASD overall severity (CaGI-S), and degree of interruption of personal time due to the child’s ASD-related issues (Item 1 CGSQ-SF7). *Note* **a**–**d** horizontal bars show medians, boxes show interquartile ranges, whiskers show Tukey intervals, *p* values are from Kruskal–Wallis tests. *AIM* Autism Impact Measure, *ASD* autism spectrum disorder, *CaGI-S* Caregiver-reported Global Impression of Severity Autism survey, *CGSQ-SF7* Caregiver Strain Questionnaire-Short form 7, *ER* emergency room

Poor child sleep quality was associated with greater impact of ASD symptoms (AIM score), higher caregiver strain scores, greater caregiver-reported ASD severity, and worse caregiver sleep quality (*p* < 0.01; Figs. 3a–d). Multiple correspondence analysis of ASD-related major adversities, together with ASD severity and caregiver time strain showed that caregivers who reported that the child eloped, or the child was non-verbal, also reported higher levels of ASD severity and higher degree of strain (Fig. 3e). Results from an additional multiple correspondence analysis on the seven items of the CGSQ-SF7 showed that all strain types were highly consistent (i.e., caregivers who responded “Very much” for one item tended to reply similarly to other items [Fig. S2]).

### Hypothesized Relationships

We analyzed the data using path mediation analysis for which we proposed models that describe the way that those variables are interrelated (Fig. 1 and Figs. S1a–b). This analysis allowed us to estimate the effects that are direct (non-mediated) or due to intermediate variables (mediated) and to assess how consistent hypothesized pathways of competing models were with empirical data.

### Direct Effects

#### Effects on Child Sleep Quality

**Fig. 4:**
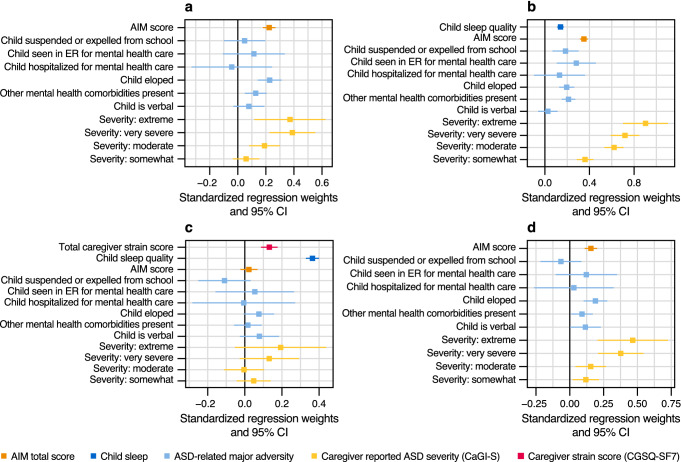
Effect of ASD core symptom impact, ASD-related major adversities, and caregiver-reported ASD severity, on child sleep quality, caregiver strain, and caregiver sleep quality. **a** Effects on child sleep quality; **b** effects on caregiver strain; **c** effects on caregiver sleep quality adjusted for caregiver strain and child sleep quality; **d** effects on caregiver sleep quality (not adjusted for caregiver strain and child sleep quality) from a multivariable linear model. *Note* effect estimates are standardized regression weights adjusted for child, caregiver, and household characteristics (as in Table 1); values over 0 correspond to a worsening of outcomes (e.g., worse sleep quality or greater caregiver strain). *AIM* Autism Impact Measure, *ASD* autism spectrum disorder, *CaGI-S* Caregiver-reported Global Impression of Severity Autism survey, *CGSQ-SF7* Caregiver Strain Questionnaire-Short form 7, *CI* confidence interval, *ER* emergency room

Male children, shorter time since ASD diagnosis, and greater household income were associated with better child sleep quality (Fig. S3). Older child age, use of medication for ASD, and large household size (≥ 5) were associated with poor child sleep quality. Families in the northeast region of the U.S. showed significantly poorer child sleep quality compared with the reference category, the west region (Fig. S3). After adjusting for all child and family demographic factors we found that caregiver impression of severity (moderately to extremely), ASD-related major adversities (mental comorbidities, child eloped), and greater impact of ASD core symptoms (AIM score) were strongly related to poor child sleep quality (*p* < 0.01; Figs. 4a, S3).

#### Effects on Caregiver Strain

Caregivers with any type of employment (compared with unemployed) or living in micropolitan areas (compared with metropolitan) had significantly less strain (*p* < 0.05; Fig. S4). Use of medication for ASD and higher caregiver age were related to greater caregiver strain (*p* < 0.05; Fig. S4). After adjusting for all demographic factors, strain markedly worsened with incremental severity from “Somewhat” to “Extremely” severe (*p* < 0.01; Fig. 4b). ASD-related major adversities such as child eloped, other mental comorbidities, child seen in ER due to mental health issues, or child suspended/expelled from school, were noticeably related to greater caregiver strain (*p* < 0.01; Fig. 4b). Increased ASD symptom impact (higher AIM scores) was related to greater caregiver strain (*p* < 0.01; Fig. 4b). In addition, independent from the effect of all ASD severity proxies above, poor child sleep quality negatively affected caregiver strain (*p* < 0.01; Fig. 4b). Regarding our proposed model in Fig. 1, these findings confirm the existence of direct effects of ASD severity (caregiver impression, ASD core symptom impact, ASD-related major adversities) and child sleep quality on caregiver strain.

#### Effects on Caregiver Sleep Quality

Household income was the most important driver among all child, caregiver, and household demographic factors on caregiver sleep quality. Higher income was markedly and incrementally related to improvement of caregiver quality of sleep (*p* < 0.01; Figs. S5a–c). Results from our multivariable model showed no statistical evidence for an effect of any of the three ASD components studied (*p* > 0.05; Fig. 4c), meaning that the child’s ASD severity (impact of ASD symptoms, caregiver impression, ASD-related major adversities) were all independent from caregiver sleep quality after adjusting for demographic factors, child sleep quality, and caregiver strain. These results suggest: (1) no evidence of direct effects of ASD severity on caregiver sleep quality, and (2) the conditional independence assumptions of our graphical model in Fig. 1 were verified (each of the three ASD components were independent from caregiver quality of sleep, adjusted for demographics, child sleep quality, and caregiver strain).

Conversely, poor child sleep quality and caregiver strain, adjusted for all other variables, were markedly related to worse caregiver sleep quality (*p* < 0.01; Fig. 4c). Child sleep quality appeared to be the most important direct effect on caregiver sleep quality (largest standardized weight; Figs. 4c, S5a). Without adjustment for child sleep quality, our ASD severity proxies were significantly associated with caregiver sleep quality (*p* < 0.05; Fig. S5b), suggesting that the effects of ASD on caregiver sleep quality were potentially mediated by child sleep quality. Similarly, not adjusting for both child sleep quality and caregiver strain shows that all ASD severity proxies were clearly associated with worse caregiver sleep quality (*p* < 0.01; Figs. 4d, S5c), suggesting that ASD effects on caregiver sleep quality were mediated by both child sleep quality and caregiver strain.

### Mediation Analysis

In order to confirm the role of intermediary variables we conducted a mediation analysis (VanderWeele, 2015). Table 3 shows indirect effect estimates and summary statistics for all pathways from ASD severity (AIM scores, ASD-related major adversities, and caregiver impression of severity) to caregiver strain and caregiver sleep quality. Impression of severity was represented by “Extremely severe” on the CaGI-S and ASD-related major adversities by the single event child eloped; results of other CaGI-S categories and ASD-related major adversities can be found in Table S1, along with posterior densities of all pathways in Figs. S6–11.

**Table 3:** Estimates of direct and indirect (mediated) effects of pathways hypothesized in our proposed model (illustrated in Fig. 1)

		95% credible interval^a^					
Effect on:	Pathway	Coef^b^	Lower	Upper	*P*(|Coef|> 0)^c^	Fraction^d^	Total mediated
CG strain	AIM score → child sleep → CG strain	0.035	0.027	0.046	**1.000**	8.5%	8.5%
	AIM score → CG strain	0.383	0.348	0.418	**1.000**	**91.5%**	
	ASD-related major adversities^e^ → child sleep → CG strain	0.035	0.021	0.05	**1.000**	13.9%	13.9%
	ASD-related major adversities → CG strain	0.213	0.145	0.281	**1.000**	**86.1%**	
	ASD severity^f^ → child sleep → CG strain	0.047	0.01	0.087	**0.994**	5.6%	5.6%
	ASD severity → CG strain	0.791	0.589	0.99	**1.000**	**94.4%**	
CG sleep	AIM score → child sleep → CG sleep	0.088	0.071	0.107	**1.000**	55.5%	
	AIM score → child sleep → CG strain → CG sleep	0.005	0.003	0.007	**1.000**	2.8%	**89.1%**
	AIM score → CG strain → CG sleep	0.049	0.032	0.067	**1.000**	30.8%	
	AIM score → CG sleep	0.017	–0.028	0.063	0.771	10.9%	–
	ASD-related major adversities → child sleep → CG sleep	0.086	0.055	0.119	**1.000**	45.7%	
	ASD-related major adversities →child sleep → CG strain → CG sleep	0.004	0.002	0.007	**1.000**	2.3%	**62.2%**
	ASD-related major adversities → CG strain → CG sleep	0.027	0.016	0.041	**1.000**	14.2%	
	ASD-related major adversities → CG sleep	0.071	–0.011	0.153	0.956	37.8%	–
	ASD severity → child sleep → CG sleep	0.118	0.026	0.212	**0.994**	26.7%	
	ASD severity → child sleep → CG strain → CG sleep	0.006	0.001	0.012	**0.994**	1.3%	**50.7%**
	ASD severity → CG strain → CG sleep	0.1	0.061	0.148	**1.000**	22.7%	
	ASD severity → CG sleep	0.218	–0.025	0.46	0.961	49.3%	–

*AIM* Autism Impact Measure, *ASD* autism spectrum disorder, *CaGI-S* Caregiver-reported Global Impression of Severity Autism survey, *CG* caregiver, *CGSQ-SF7* Caregiver Strain Questionnaire-Short form 7, *Coef* standardized regression weight

^a^Credible intervals, i.e., 2.5% and 97.5% percentiles of the posterior distribution

^b^Posterior median of the standardized weights

^c^Posterior probability: *P*(|Coef|> 0 | data)

^d^Fraction of the effect that is mediated/not mediated

^e^Child eloped

^f^“Extremely severe” on the CaGI-S

#### Effects on Caregiver Strain

We found evidence (certainty > 99%) that child sleep quality mediated the effects of ASD (impact of core symptoms, ASD-related major adversities, caregiver impression of severity) on caregiver strain (Tables 3, S1; Fig. S7). However, these mediated effects represent only a small fraction of the total effect: 8.5%, 13.9%, and 5.6% of the effects of degree of ASD symptoms, ASD-related major adversities, and impression of severity respectively, are mediated effects (Tables 3, S1).

#### Effects on Caregiver Sleep Quality

We found evidence (certainty > 99%) that the effects of child’s ASD severity (as AIM scores, ASD-related major adversities, and caregiver impression of severity) were mediated by either child sleep quality or caregiver strain or sequentially by both (Tables 3, S1; Figs. S9–S11). The exception were two pathways of child in ER due to mental health problems (Table S1; Figs. S9–S10). Moreover, most of the effects of ASD severity on caregiver sleep were mediated: 89.1% of the effects coming from ASD core symptoms were mediated, child sleep quality was the most important mediator (55.5% + 2.8% of these effects were mediated by child sleep; Tables 3 and S1 and Figs. S9–S10). Similarly, effects of ASD-related major adversities on caregiver sleep were mainly mediated (e.g., 62.2% of effects when child eloped were mediated) with child sleep quality as the most important mediator (Tables 3, S1). Severity reported by caregivers appeared to be related to caregiver sleep through either child sleep quality or caregiver strain (Tables 3, S1; Figs. S9–S11).

Our results confirmed our finding above that there is no evidence that the child’s ASD severity has direct effects on caregiver sleep as the 95% credible intervals corresponding to all ASD direct effects on caregiver sleep include 0 (Tables 3, S1).

## Discussion

This study provided the unique opportunity to elucidate the impact of ASD on families demonstrating the relationship between a child’s ASD severity and caregiver strain, and caregiver and child sleep quality. First, we identified child, caregiver, and household characteristics that were associated with worse child sleep quality: older child age, female gender, longer time since diagnosis, use of medication for ASD, living in the northeast (over west) U.S. region, lower household income, and household size of ≥ 5. Increased caregiver strain was associated with child use of medication for ASD, living in a metropolitan area (over micropolitan), increased caregiver age, and caregiver unemployment. Better caregiver sleep quality correlated with incremental household income.

Our results further suggest that the degree of the child’s ASD severity remains a prominent factor in the quality of life of both the child and the caregiver. In addition to the characteristics listed above, we found that increased ASD severity captured as impact and frequency of ASD core symptoms (AIM score), caregiver impression of severity (CaGI-S), and occurrence of ASD-related major adversities results in reduced child sleep quality. Studies have shown that increased ASD severity increases the likelihood of sleep problems and can worsen the severity of sleep problems (Adams et al., 2014). Furthermore, although sleep disturbances are not an etiological factor of ASD, they can be a consequence that reinforces the core symptoms of ASD (Deliens & Peigneux, 2019; Tudor et al., 2012). Based on the current literature, it remains difficult to establish the directionality of the association (Deliens & Peigneux, 2019), and to verify this feedback loop longitudinal data are required.

We also found that the child’s ASD severity and child sleep quality led to increased caregiver strain, and that the child’s sleep problems mediate part of the effects of ASD severity on caregiver strain. To our knowledge, this finding has not been previously reported. The ASD-related adversities that we found to be strongly related to caregiver strain were the existence of other mental health comorbidities, child eloped, child in ER for mental health problems, and child suspended from school. Eloping concerns have previously been shown to increase worry and anxiety for parents and negatively impact household routines (Anderson et al., 2012). Providing family-based support that targets parents, for example training on managing the specific stresses of caregiving and avoiding maladaptive coping (Lee et al., 2019; Rodriguez et al., 2019), could help to alleviate some of the strain associated with worry or anxiety. Finally, ASD effects on caregiver sleep quality were predominantly mediated by either a reduction of child sleep quality, or by increased caregiver strain, or by both. Indeed, we did not find evidence that ASD severity reduces caregiver sleep quality, unless it was through worsening of child sleep quality or through increased caregiver strain, or via a combination of both. Previous research suggests that parents of children with ASD who sleep poorly have poor quality sleep (Goldman et al., 2014; Lee, 2013). Positive associations have been found for mothers of ASD children who sleep well and their child’s total sleep time (Goldman et al., 2014). Additionally, poor parental sleep quality is linked to inability to cope with stressors and dysfunctional parenting (McQuillan et al., 2019). Our findings align with previous research showing that caregiver stress is a predictor of poor caregiver sleep (Gallagher et al., 2010) and, together with other studies (Dykens et al., 2014; Khanna et al., 2011; Lindly et al., 2021; Rodriguez et al., 2019), emphasize the importance of providing support directly to caregivers to reduce caregiver stress and strain, and positively affect caregiver sleep and quality of life.

To our knowledge, there is no prior evidence that the impact of child ASD severity on caregiver sleep is essentially mediated by child sleep problems and/or increased caregiver stress. Low sleep quality and short sleep duration (either due to difficulty falling asleep or early morning wakening) are reported to be the most frequent sleep issues for children with ASD (Carmassi et al., 2019), although sleep disorders in children with ASD are heterogenous (Deliens & Peigneux, 2019). Our study looked at overall sleep quality but considering the above-mentioned diversity of sleep disorders it will be of practical importance for future research to further investigate the relationships between different sleep disorders and caregiver strain. A better understanding of which child sleep disturbances cause increases in caregiver strain could help to prioritize specific interventions that would have the highest positive impact for families.

We found that the three ways with which we captured the child’s ASD severity each had separate downstream effects on all outcome variables directly or indirectly (degree of ASD core symptoms, caregiver impression of severity, and occurrence of ASD-related major adversities). This indicates that they consistently assess the severity of ASD; however, they also seemed to have elements that translated separately into downstream changes in child sleep quality, caregiver strain, and caregiver sleep quality. Both caregiver impression of severity and AIM scores, a measure of frequency and impact of core ASD symptoms, consistently influenced all outcomes directly or indirectly. The ASD-related major adversities such as if the child eloped and presence of other mental health comorbidities had effects on all outcomes (directly or indirectly) while suspension from school and hospitalization in ER due to mental health comorbidities strongly correlated with increased caregiver strain.

Our results are based on the evaluation of a model that describes the pathways through which increased ASD severity affects child sleep quality, caregiver strain, and caregiver sleep quality. All implicit conditional independence assumptions, including those of competing models (Figs. S1a–b), were verified. Alternative models were discarded because of inconsistency with empirical data. For instance, the model in Fig. S1a states that child sleep is independent from caregiver strain after controlling for all ASD severity proxies and demographics, which was not supported by our data. Similarly, we discarded the model in Fig. S1b because it states that (1) all ASD severity proxies have direct effects on caregiver sleep (regardless of child sleep and caregiver strain), and (2) caregiver sleep is independent from strain after controlling for all ASD severity proxies, child sleep, and demographics.

Limitations of this study include the cross-sectional design, reliance on caregiver-reported assessments of their children, and accuracy of respondent recall. In this study we did not account for sleep interventions (due to lack of information) when relating sleep quality with the other parameters of the model and due to the cross-sectional design of the sleep quality assessment, our results represent a caregiver-reported mean sleep quality, which assumes that poor sleepers consistently sleep badly every night. Abnormalities in sleep are not always perceived by the children themselves or by their caregivers and therefore would not be identified through questionnaires (Deliens & Peigneux, 2019), the methodology used in this study. We used a simple, self-designed question to capture sleep quality since other validated questionnaires would have made the overall study survey too extensive. It was not possible with cross-sectional data to study potential feedback loop effects between ASD severity and child sleep, or between caregiver strain and caregiver sleep quality. Our analysis did not consider subgroups of children with ASD (for example those who may be cognitively impaired) because of incomplete data.

Strengths of the study include the large number of respondents across all four U.S. regions, the use of validated outcome measures (AIM and CGSQ-SF7), and assessment of objective and subjective ASD severity. The cohort was generally representative of children with ASD in the U.S.; demographic and baseline characteristics were similar with those reported in the 2016 National Survey of Children’s Health (Data Resource Center for Child & Adolescent Health, 2018). The statistical methodology allowed us to control for confounding and to disentangle the role of mediators and provided insight on how variables are interrelated. Finally, the data collection was from a large sample not linked to a specific provider or network of centers and these data will be made available via SPARK and will be linkable to other data collected from the same cohort.

The eligibility criteria age range for children of participants was selected for two reasons. First, AIM is designed to measure subtle changes in core ASD symptoms in individuals aged 3–18 years old (Kanne et al., 2014; Mazurek et al., 2018). Second, SPARK generally collects data on caregivers up to the child’s 18^th^ birthday, after which the dependent can consent to participate themselves, or if the caregiver is the custodial guardian, the caregiver can agree to participate on behalf of the dependent. In order to ensure caregiver availability to participate in our study, we limited the age of children with ASD to 3–17 years.

In conclusion, the degree of ASD severity markedly affects caregiver strain and children’s sleep quality in addition to important demographic, geographic, and socioeconomic characteristics. Child sleep problems play a role as a minor mediator on increasing caregiver strain. The degree of ASD severity did not influence caregiver sleep quality unless it was through worsening of child sleep quality or greater caregiver strain. Our results suggest that interventions aimed not only at ASD itself but also at reducing caregiver strain or improving child sleep quality could have a positive impact for caregivers and on the care of children with ASD. Furthermore, ASD-related major adversities such as: the child eloped, child in emergency hospitalization or mental problems, child expelled from school, and others, which can affect child sleep, caregiver strain, and sleep directly or indirectly. This allows identification of areas of support for caregivers to improve their abilities to cope with ASD-specific stressors, facilitate a reduction in caregiver strain, and in turn foster increased family functioning.

## Supplementary Information

Below is the link to the electronic supplementary material.

Supplementary file1 (PDF 800 KB)

## Data Availability

For up-to-date details on Roche's Global Policy on the Sharing of Clinical Information and how to request access to related clinical study documents, see here: https://go.roche.com/data_sharing. Request for the data underlying this publication requires a detailed, hypothesis-driven statistical analysis plan that is collaboratively developed by the requestor and company subject matter experts. Such requests should be directed to datarequest.autism@roche.com for consideration. Anonymized records for individual patients across more than one data source external to Roche cannot, and should not, be linked due to a potential increase in risk of patient re-identification.
