# Supplementary material for: Effect of Children’s Autism Spectrum Disorder Severity on Family Strain and Sleep Quality: A Cross-Sectional Online Survey in the U.S
Source: J Autism Dev Disord. 2022 Feb 3;53(5):1795–808. doi: 10.1007/s10803-022-05457-7 (PMC10123033; doi:10.1007/s10803-022-05457-7)
Supplement: Supplementary file 1 — Supplementary file1 (PDF 800 KB) [file 10803_2022_5457_MOESM1_ESM.pdf]

Supplementary Information

Effect of Children's Autism Spectrum Disorder Severity on Family Strain and Sleep Quality – A Cross-Sectional Online Survey in the U.S.

Journal of Autism and Developmental Disorders

[Author names removed for blind peer review]

Contents

Supporting Information Figs. S1–S11 Page 2

Supporting Information Table S1 Page 15

Supporting Information Fig. 1 Directed acyclic graphs (DAG) with alternative hypothesized relationships between severity of child's autism spectrum disorder (ASD) (Caregiver impression of severity, ASD core symptoms, ASD-related major adversities) with child sleep quality, caregiver (CG) strain, and caregiver sleep quality. a) ASD severity directly affects child sleep and CG strain but not CG sleep. Child sleep does not affect CG strain directly and cannot be a mediator of severity. ASD affects CG sleep only through child sleep or CG strain. b) ASD directly affects all three: child sleep, CG strain, and CG sleep. CG strain does not have a direct effect on CG sleep, i.e., controlling for all variables in the DAG, CG strain, and CG sleep are independent

a)

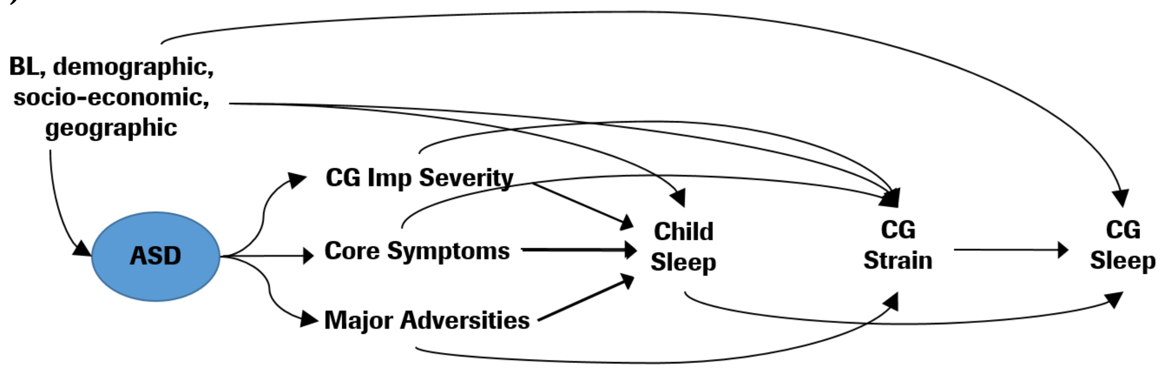

b)

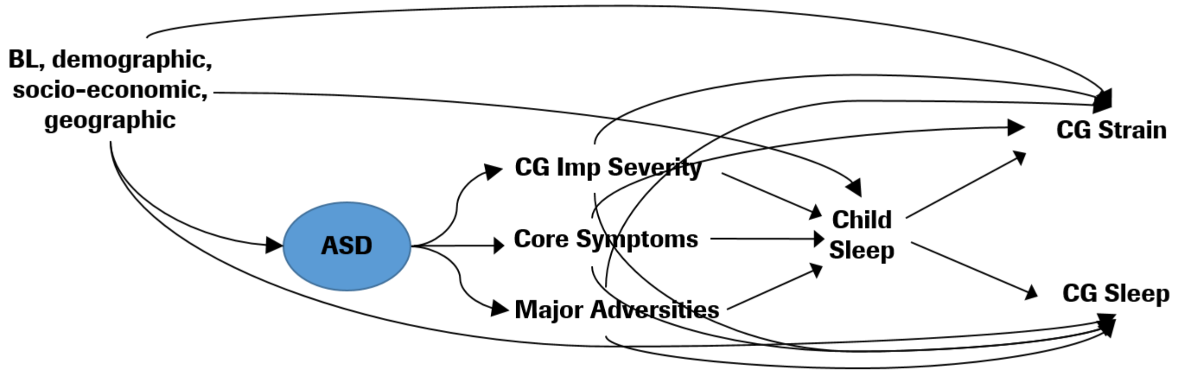

Note. Baseline demographics, socioeconomic, and geographic characteristics for household, caregiver, and child.

Abbreviations: *ASD* autism spectrum disorder, *BL* baseline, *CG* caregiver, *imp* impression.

Supporting Information Fig. 2 Multiple correspondence analysis conducted on the seven types of caregiver strain in the Caregiver Strain Questionnaire-Short Form 7 (CGSQ-SF7)

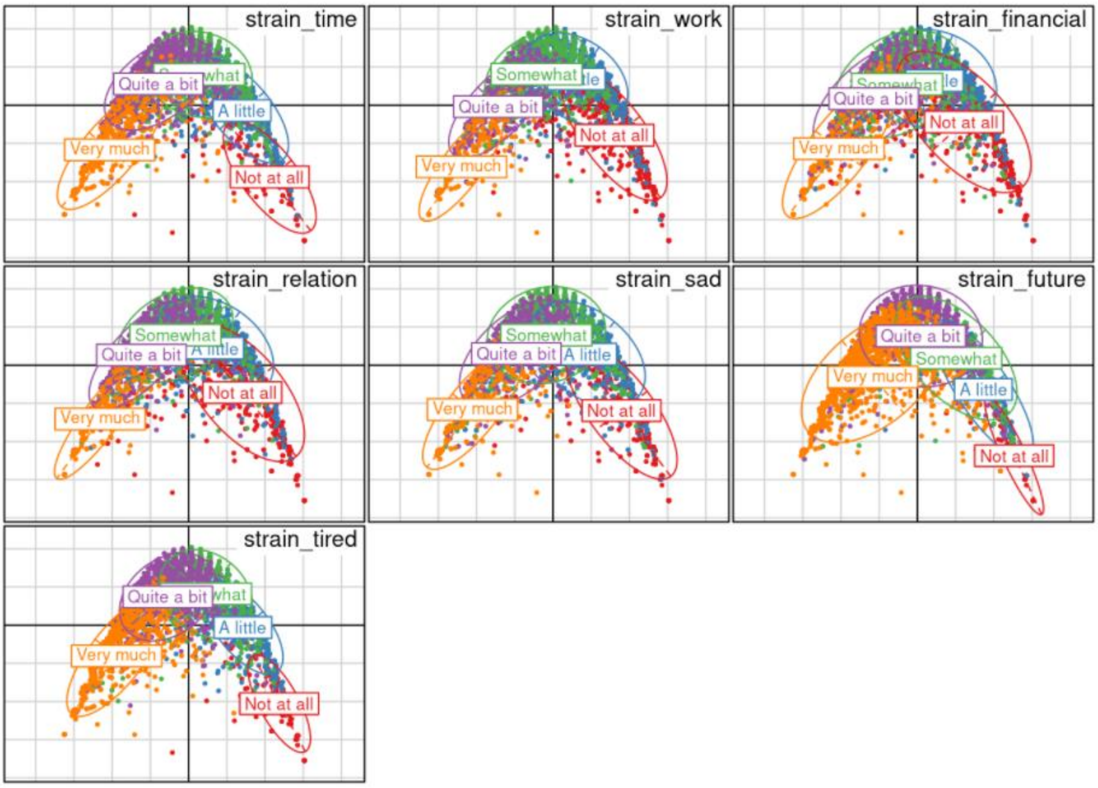

Note. In response to the question 'In the past month and resulting from your child's problems, how much of a problem was':

strain_time = Interruption of personal time

strain_work = Missing work or neglecting other duties

strain_finantial = Financial strain for your family

strain_relation = Disruption or upset of relationships within the family

strain_sad = Feeling sad or unhappy

strain_future = Worried about your child's future

strain_tired = Feeling tired or strained.

Abbreviation: *CGSQ-SF7* Caregiver Strain Questionnaire-Short form 7.

Supporting Information Fig. 3 Standardized regression weights of a multivariable model of child quality of sleep as a function of ASD symptoms (AIM score), ASD-related major adversities (child is verbal, has other mental comorbidities, eloped, hospitalized for mental problems, seen in ER due to mental problems, suspended/expelled from school), ASD severity (CaGI-S) adjusted for child, caregiver, and household demographic characteristics. A positive coefficient indicates reduction in the quality of sleep. A negative coefficient indicates an improvement

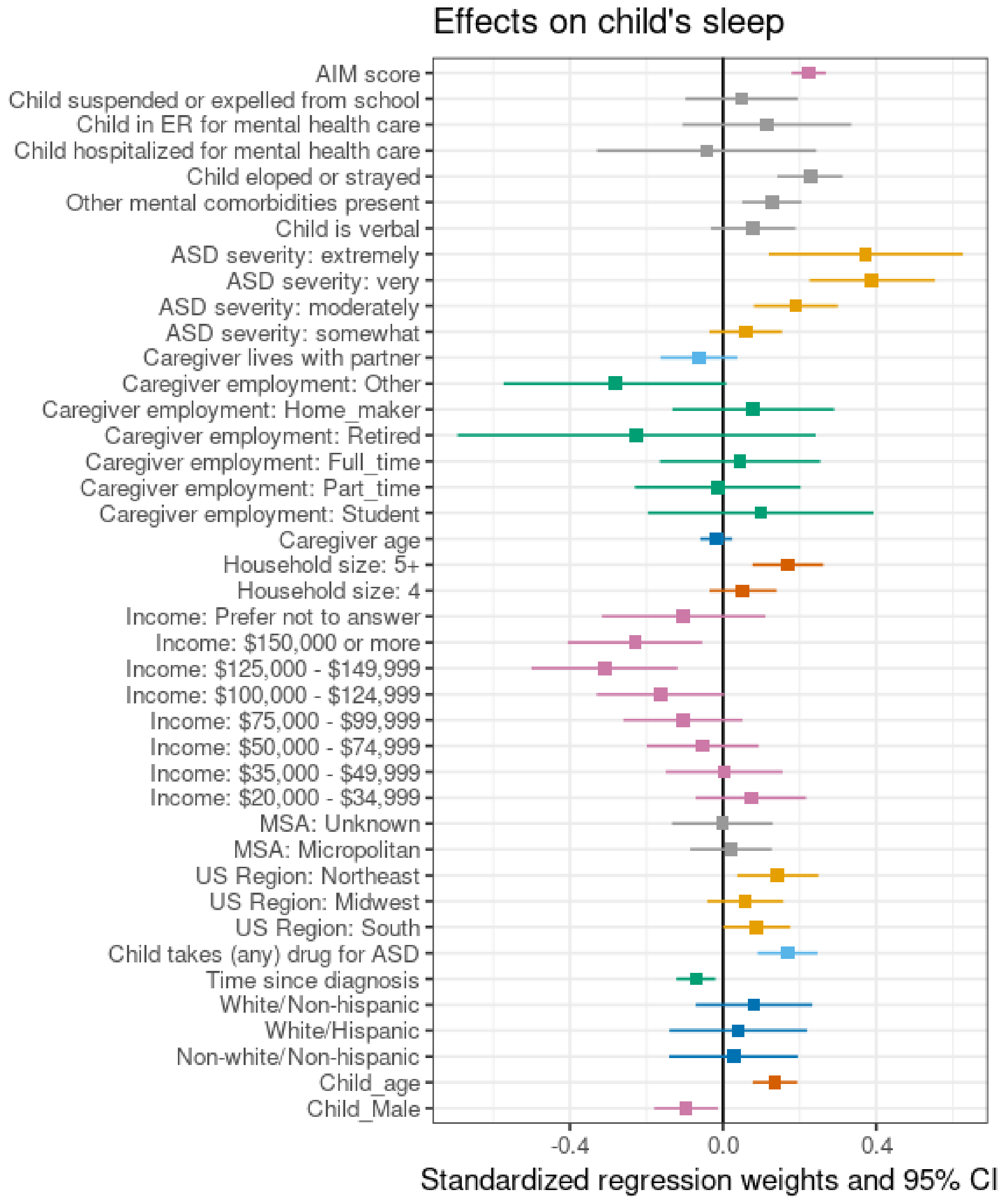

Note. Demographic variables and household characteristics drawn from the 2017 baseline survey.

Abbreviations: *AIM* Autism Impact Measure, *ASD* autism spectrum disorder, *CaGI-S* Caregiver-reported Global Impression of Severity Autism survey, *CG* caregiver, *CI* confidence interval, *ER* emergency room, *MSA* metropolitan service area, *US* United States.

Supporting Information Fig. 4 Standardized regression weights of a multivariable model of total caregiver strain score (CGSQ-SF7) as a function of: ASD symptoms (AIM score), ASD-related major adversities (child is verbal, has other mental comorbidities, eloped, hospitalized for mental problems, seen in ER due to mental problems, suspended/expelled from school), ASD severity (CaGI-S), and child quality of sleep, adjusted for child, caregiver, and household characteristics. A positive coefficient indicates worse strain, a negative coefficient indicates improvement

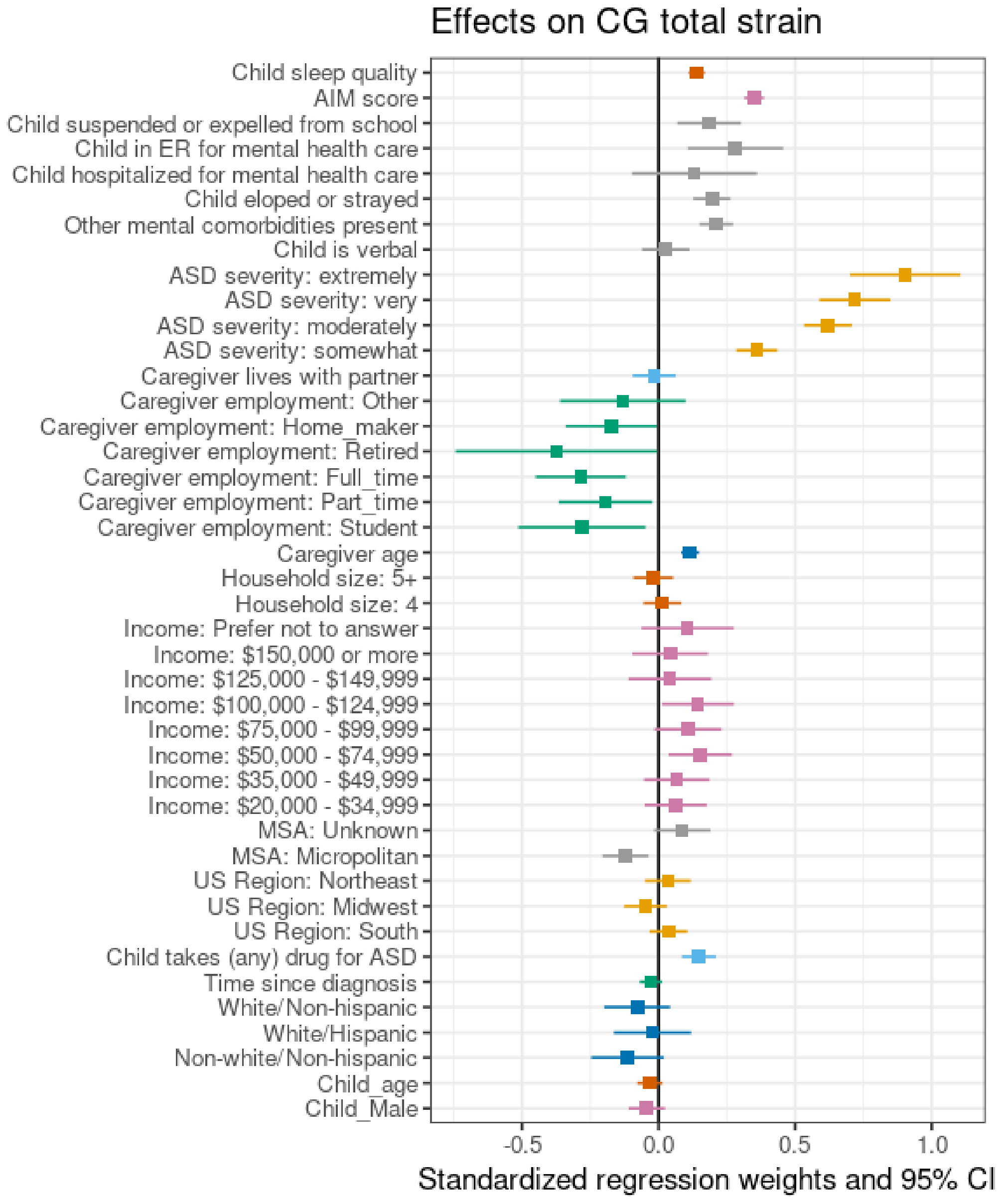

Note. Demographic variables and household characteristics drawn from the 2017 baseline survey.

Abbreviations: *AIM* Autism Impact Measure, *ASD* autism spectrum disorder, *CaGI-S* Caregiver-reported Global Impression of Severity Autism survey, *CG* caregiver, *CGSQ-SF7* Caregiver Strain Questionnaire-Short form 7, *CI* confidence interval, *ER* emergency room, *MSA* metropolitan service area, *US* United States.

Supporting Information Fig. 5 (a) Standardized regression weights of a multivariable model of caregiver quality of sleep as a function of: ASD symptoms (AIM score), ASD-related major adversities (child is verbal, has other mental comorbidities, eloped, hospitalized for mental problems, seen in ER due to mental problems, suspended/expelled from school), ASD severity (CaGI-S), child quality of sleep, and caregiver strain score (CGSQ-SF7), adjusted for child, caregiver, and household characteristics. A positive coefficient indicates reduction of the quality of sleep. A negative coefficient indicates an improvement

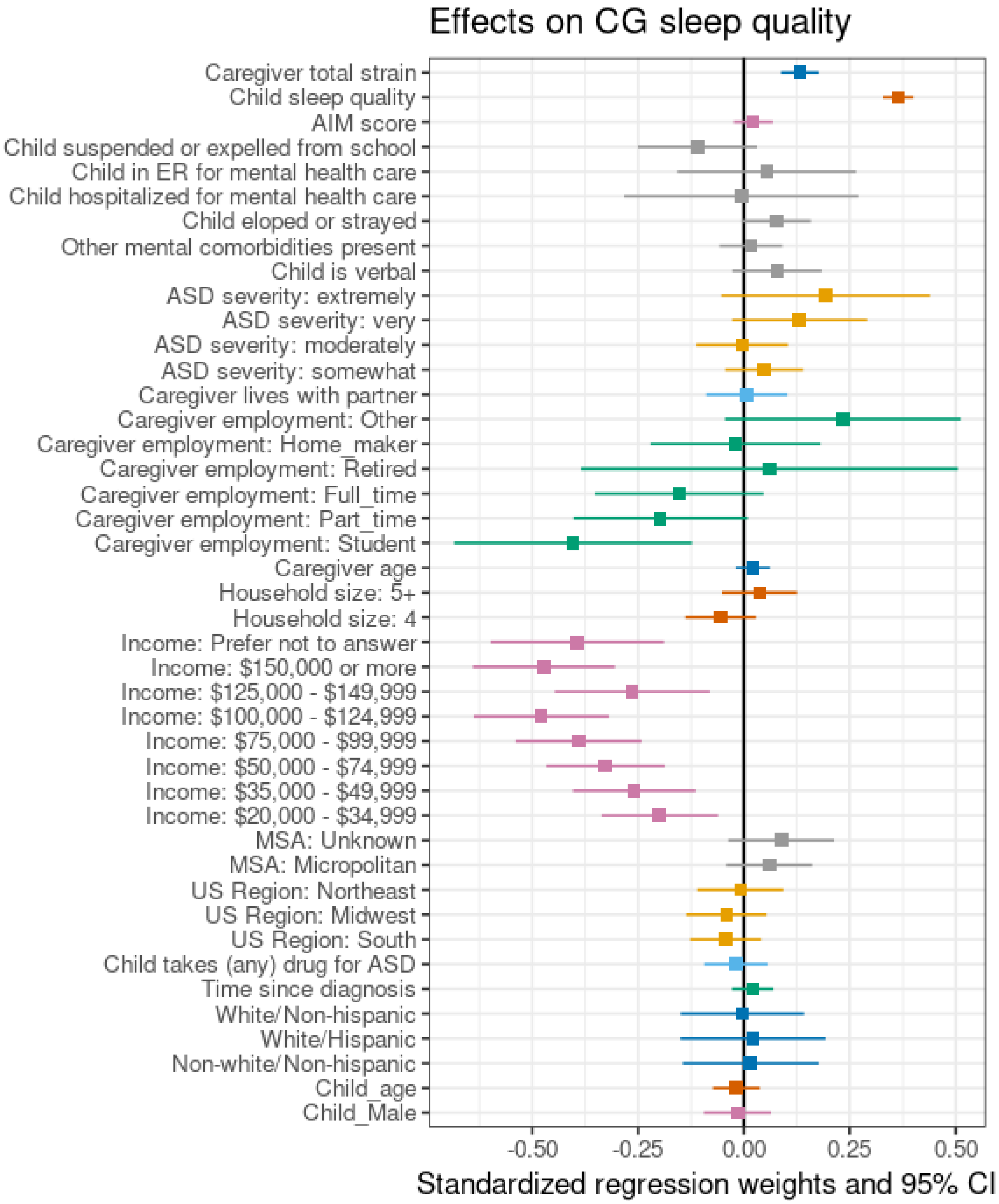

Note. Demographic variables and household characteristics drawn from the 2017 baseline survey.

Supporting Information Fig. 5 (b) Same model as panel (a) but without adjusting for child quality of sleep

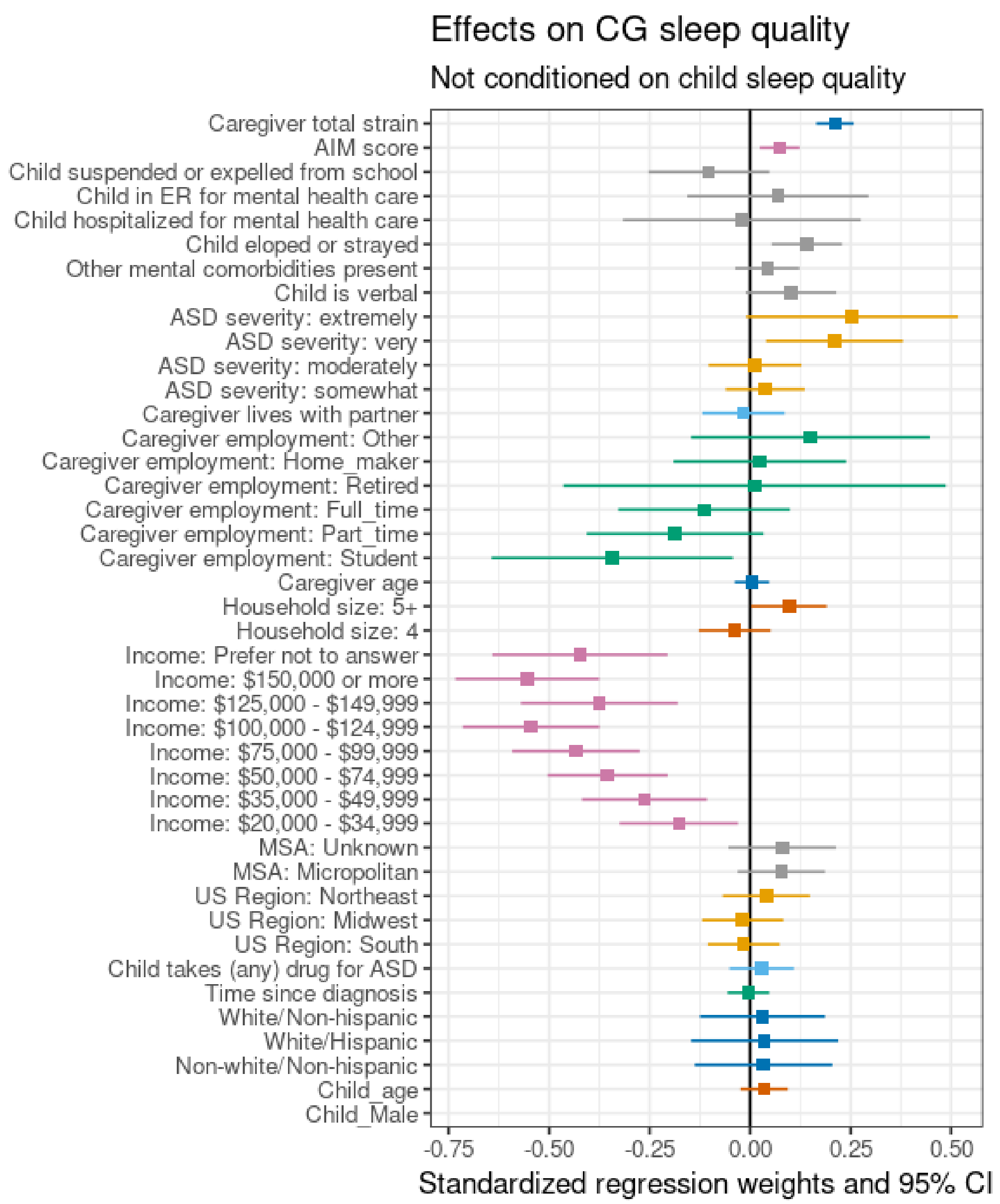

Note. Demographic variables and household characteristics drawn from the 2017 baseline survey.

Supporting Information Fig. 5 (c) Same model as in panel (a) but without adjusting for both child quality of sleep and caregiver strain (CGSQ-SF7)

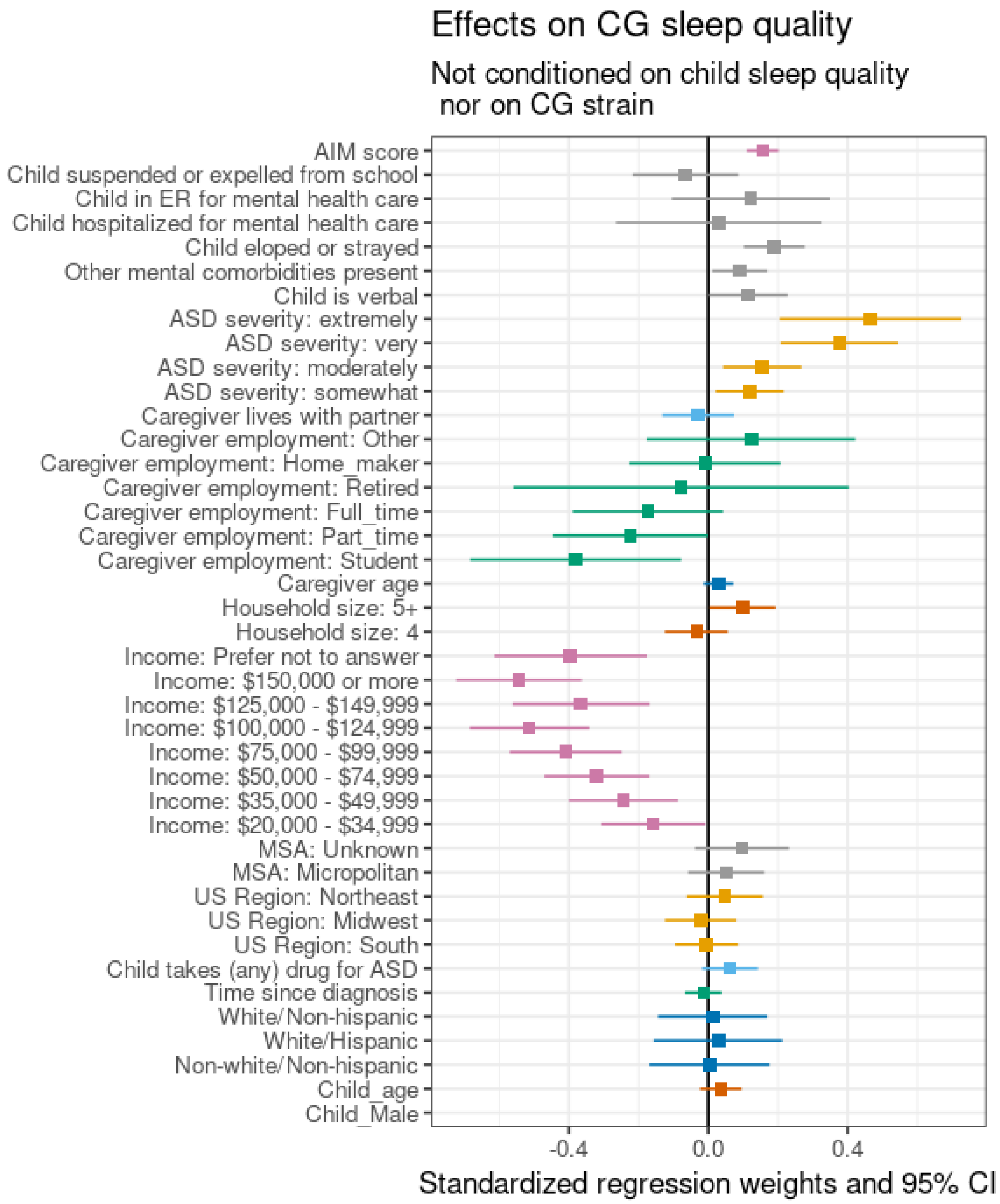

Note. Demographic variables and household characteristics drawn from the 2017 baseline survey.

Abbreviations: *AIM* Autism Impact Measure, *ASD* autism spectrum disorder, *CaGI-S* Caregiver-reported Global Impression of Severity Autism survey, *CG* caregiver, *CGSQ-SF7* Caregiver Strain Questionnaire-Short form 7, *CI* confidence interval, *ER* emergency room, *MSA* metropolitan service area, *US* United States.

Supporting Information Fig. 6 Posterior densities of the direct (not mediated) effects of: ASD-related major adversities (elope, other mental comorbidities, and ER hospitalization due to mental problems), ASD severity (moderate, very, extremely; CaGI-S), ASD symptoms (AIM score), and child quality of sleep on caregiver strain (CGSQ-SF7). Other denotes a selected variable that does not have effects on caregiver strain (caregiver living with partner). Red dashed vertical line denotes the "No-Effect" region

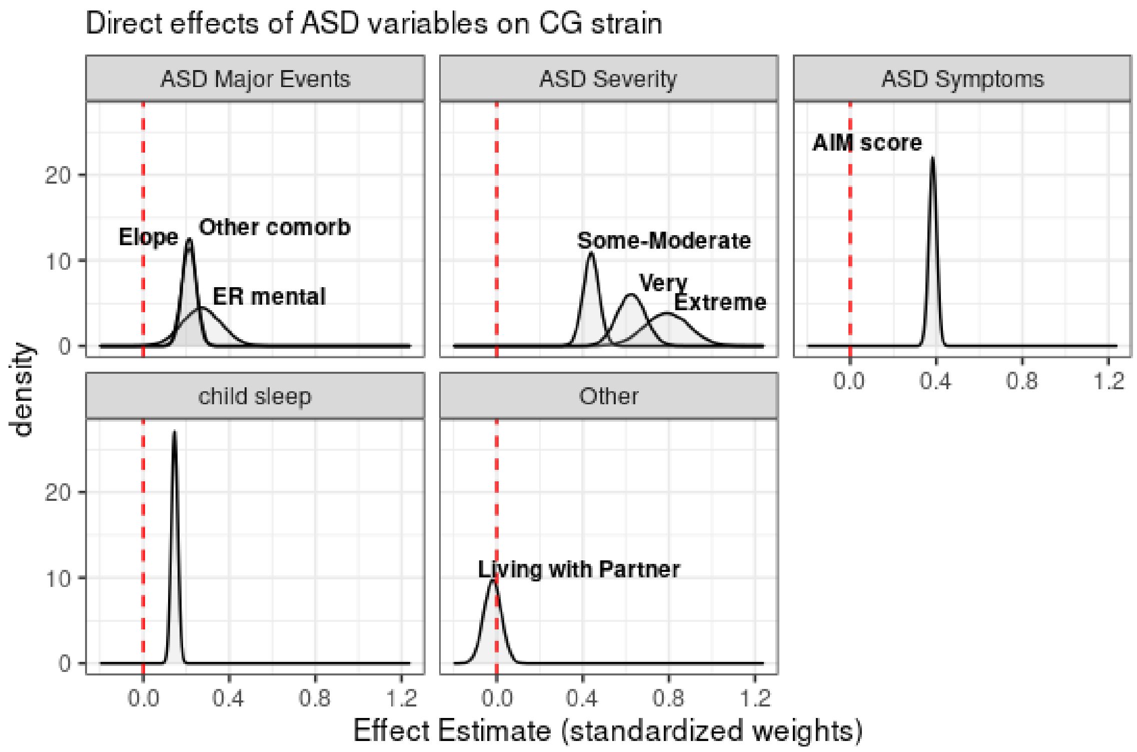

Abbreviations: *AIM* Autism Impact Measure, *ASD* autism spectrum disorder, *CaGI-S* Caregiver-reported Global Impression of Severity Autism survey, *CG* caregiver, *CGSQ-SF7* Caregiver Strain Questionnaire-Short form 7, *ER* emergency room.

Supporting Information Fig. 7 Posterior densities of indirect effects of: ASD-related major adversities (elope, other mental comorbidities, and ER hospitalization due to mental problems) ASD severity (CaGI-S), and ASD symptoms (AIM score) on caregiver strain (CGSQ-SF7) mediated by child sleep quality. Red dashed vertical line denotes the "No-Effect" region

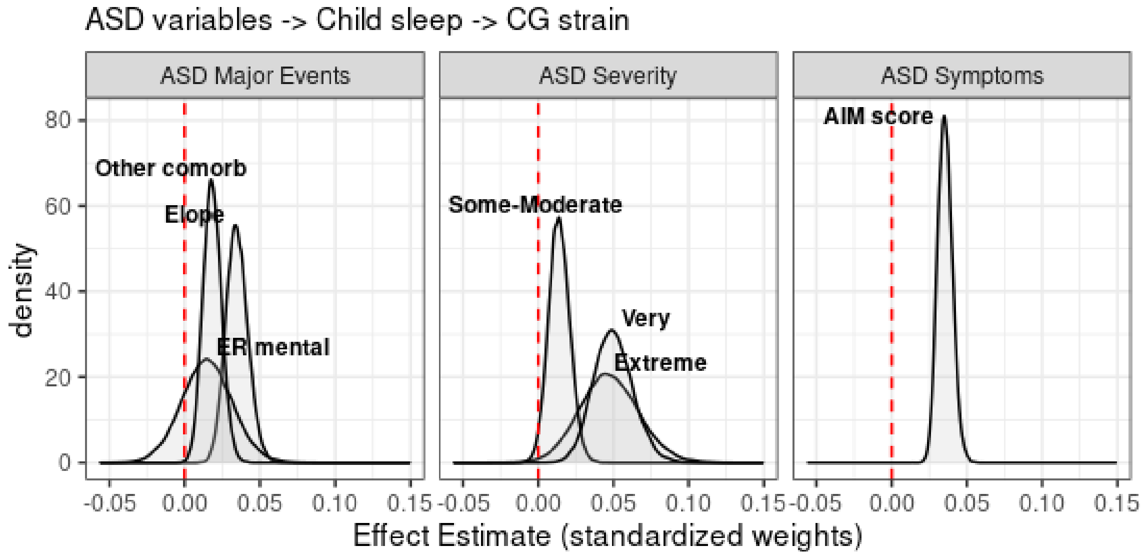

Abbreviations: *AIM* Autism Impact Measure, *ASD* autism spectrum disorder, *CaGI-S* Caregiver-reported Global Impression of Severity Autism survey, *CG* caregiver, *CGSQ-SF7* Caregiver Strain Questionnaire-Short form 7, *ER* emergency room.

Supporting Information Fig. 8 Posterior densities of the direct (not mediated) effects of: ASD-related major adversities (elope, other mental comorbidities, and ER hospitalization due to mental problems), ASD severity (moderate, very extremely; CaGI-S), ASD symptoms (AIM score), child quality of sleep, and caregiver strain (CGSQ-SF7) on caregiver sleep quality. Other denotes a selected variable that does not have effects on caregiver sleep quality (i.e., caregiver living with partner). Red dashed vertical line denotes the "No-Effect" region

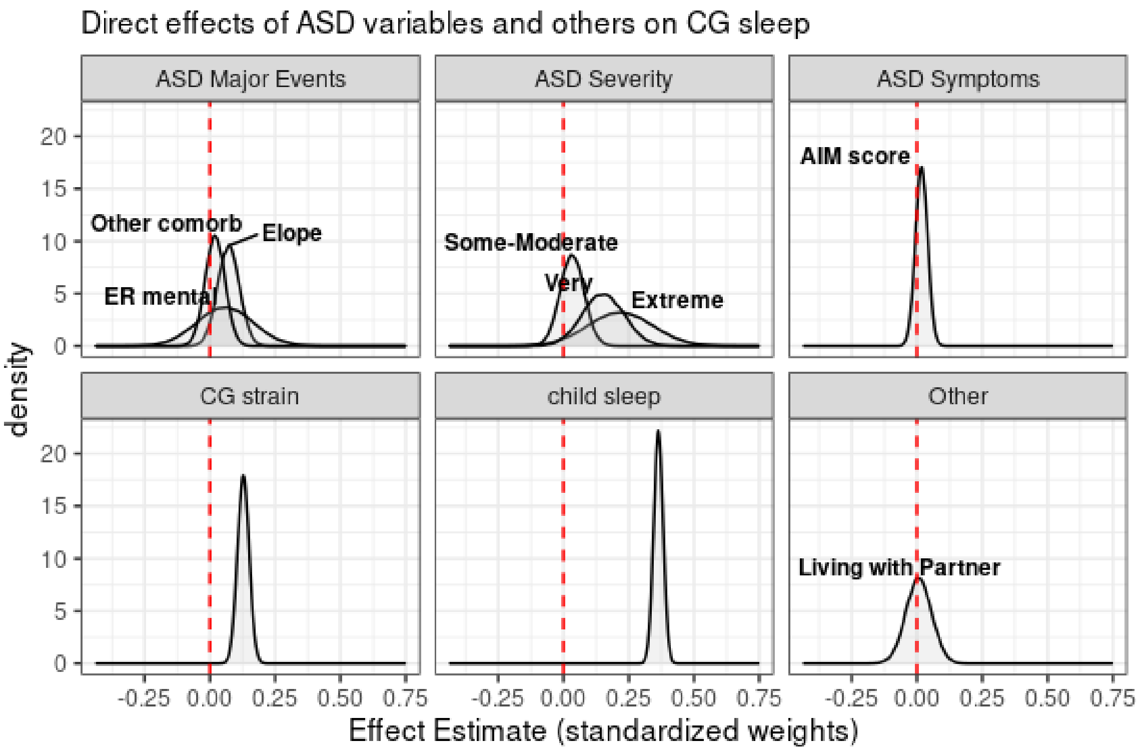

Abbreviations: *AIM* Autism Impact Measure, *ASD* autism spectrum disorder, *CaGI-S* Caregiver-reported Global Impression of Severity Autism survey, *CG* caregiver, *CGSQ-SF7* Caregiver Strain Questionnaire-Short form 7, *ER* emergency room.

Supporting Information Fig. 9 Posterior densities of indirect effects of: ASD-related major adversities (elope, other mental comorbidities, and ER hospitalization due to mental problems), ASD severity (CaGI-S), and ASD symptoms (AIM score) on caregiver sleep quality, mediated by child sleep quality independent from caregiver strain (CGSQ-SF7). Red dashed vertical line denotes the “No-Effect” region

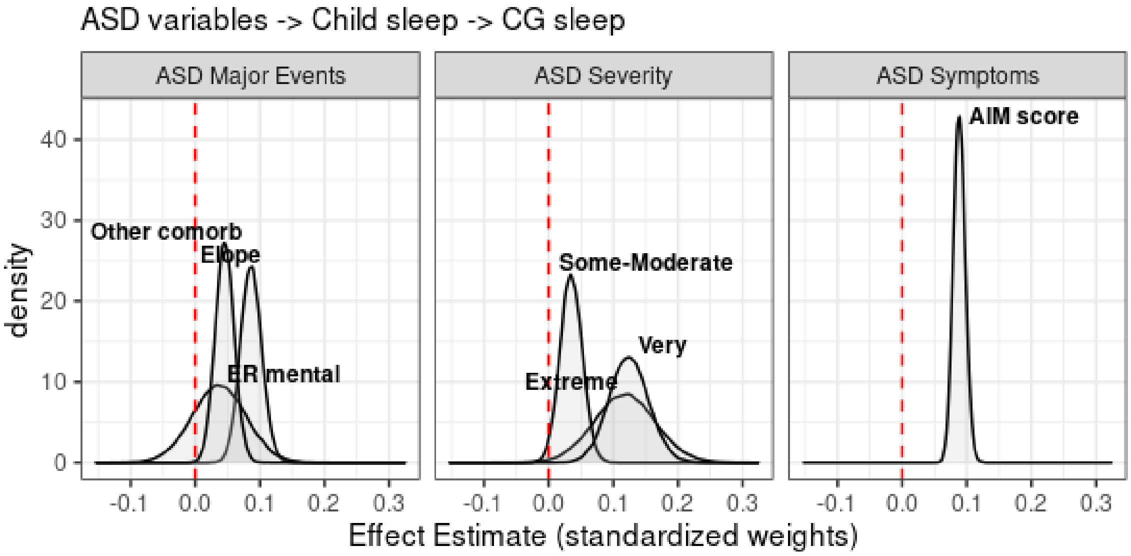

Abbreviations: *AIM* Autism Impact Measure, *ASD* autism spectrum disorder, *CaGI-S* Caregiver-reported Global Impression of Severity Autism survey, *CG* caregiver, *CGSQ-SF7* Caregiver Strain Questionnaire-Short form 7, *ER* emergency room.

Supporting Information Fig. 10 Posterior densities of indirect effects of: ASD-related major adversities (elope, other mental comorbidities, and ER hospitalization due to mental problems), ASD severity (CaGI-S), and ASD symptoms (AIM score) on caregiver sleep quality, mediated by child sleep, and caregiver strain (CGSQ-SF7). Red dashed vertical line denotes the “No-Effect” region

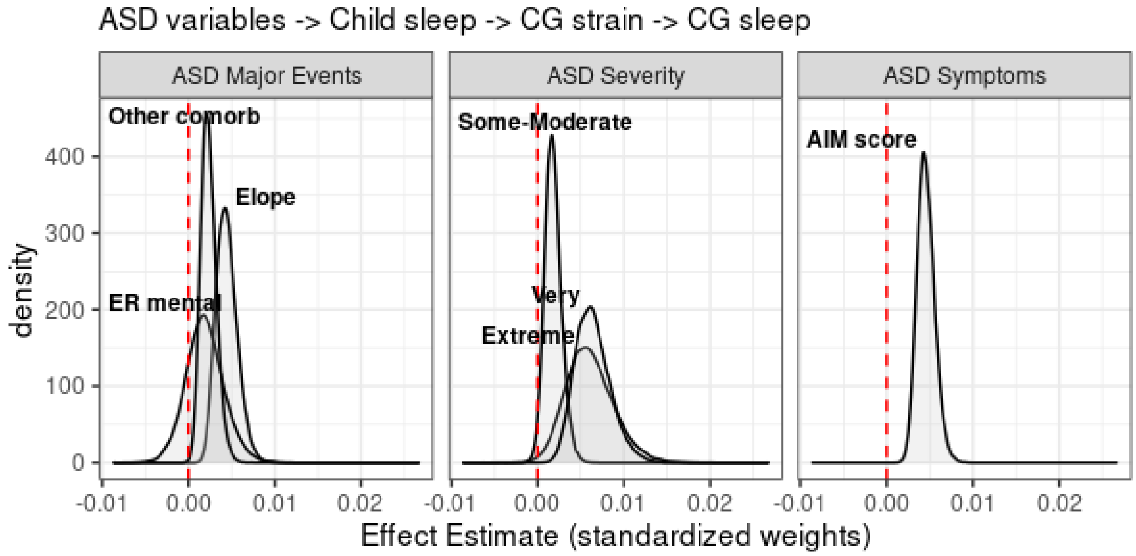

Abbreviations: *AIM* Autism Impact Measure, *ASD* autism spectrum disorder, *CaGI-S* Caregiver-reported Global Impression of Severity Autism survey, *CG* caregiver, *CGSQ-SF7* Caregiver Strain Questionnaire-Short form 7, *ER* emergency room.

Supporting Information Fig. 11 Posterior densities of indirect effects of: ASD-related major adversities (elope, other mental comorbidities, and ER hospitalization due to mental problems), ASD severity (CaGI-S), and ASD symptoms (AIM score) on caregiver sleep quality, mediated by caregiver strain (CGSQ-SF7) independent from child sleep. Red dashed vertical line denotes the "No-Effect" region

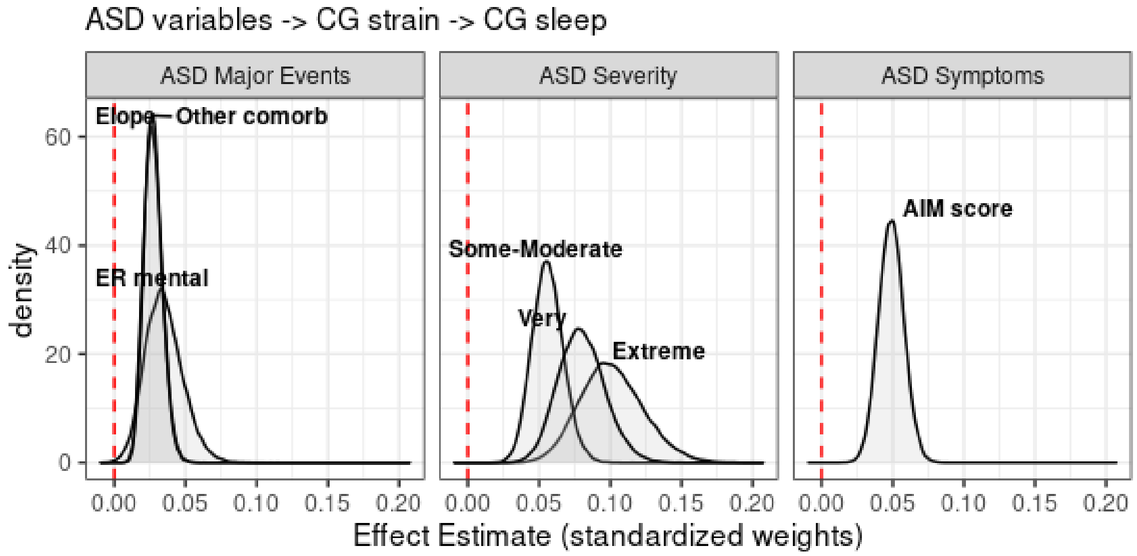

Abbreviations: *AIM* Autism Impact Measure, *ASD* autism spectrum disorder, *CaGI-S* Caregiver-reported Global Impression of Severity Autism survey, *CG* caregiver, *CGSQ-SF7* Caregiver Strain Questionnaire-Short form 7, *ER* emergency room.

Supporting Information Table 1 Summary of statistics from posterior distributions of direct and indirect effects: Posterior median, 2.5 and 97.5 percentiles, posterior probability that |parameter|>0. Fraction denotes the proportion of the total effect that goes through a given pathway

Parameter	type	from	effect_type	effect_on	Median	Lower	Upper	P_diff_0	fraction
asd.sev0.chsl.str	ASD Severity	0_some_moder	mediated	1 CG strain	0.014	0.001	0.028	0.980	0.031
b.strn[39]	ASD Severity	0_some_moder	direct	1 CG strain	0.439	0.365	0.512	1.000	0.969
asd.sev1.chsl.str	ASD Severity	1_very	mediated	1 CG strain	0.049	0.026	0.077	1.000	0.073
b.strn[40]	ASD Severity	1_very	direct	1 CG strain	0.625	0.497	0.752	1.000	0.927
asd.sev2.chsl.str	ASD Severity	2_extreme	mediated	1 CG strain	0.047	0.010	0.087	0.994	0.056
b.strn[41]	ASD Severity	2_extreme	direct	1 CG strain	0.791	0.589	0.990	1.000	0.944
asd.symp.chsl.str	ASD Symptoms	AIM	mediated	1 CG strain	0.035	0.027	0.046	1.000	0.085
b.strn[32]	ASD Symptoms	AIM	direct	1 CG strain	0.383	0.348	0.418	1.000	0.915
asd.mjev.elop.chsl.str	ASD Major Adversities	Elope	mediated	1 CG strain	0.035	0.021	0.050	1.000	0.139
b.strn[28]	ASD Major Adversities	Elope	direct	1 CG strain	0.213	0.145	0.281	1.000	0.861
asd.mjev.erhp.chsl.str	ASD Major Adversities	ER mental	mediated	1 CG strain	0.015	-0.018	0.048	0.812	0.051
b.strn[30]	ASD Major Adversities	ER mental	direct	1 CG strain	0.272	0.097	0.447	0.999	0.949
asd.mjev.otml.chsl.str	ASD Major Adversities	Other comorb	mediated	1 CG strain	0.018	0.007	0.031	0.999	0.078
b.strn[27]	ASD Major Adversities	Other comorb	direct	1 CG strain	0.214	0.153	0.275	1.000	0.922
b.strn[33]	Child sleep	Child sleep	direct	1 CG strain	0.146	0.117	0.175	1.000	1.000
asd.sev0.chsl.cgsl	ASD Severity	0_some_moder	mediated	2 CG sleep	0.035	0.002	0.069	0.980	0.277
asd.sev0.chsl.str.cgsl	ASD Severity	0_some_moder	mediated	2 CG sleep	0.002	0.000	0.004	0.980	0.014
asd.sev0.str.cgsl	ASD Severity	0_some_moder	mediated	2 CG sleep	0.056	0.036	0.079	1.000	0.446
b.cgsl[40]	ASD Severity	0_some_moder	direct	2 CG sleep	0.033	-0.056	0.124	0.766	0.263
asd.sev1.chsl.cgsl	ASD Severity	1_very	mediated	2 CG sleep	0.124	0.065	0.185	1.000	0.343
asd.sev1.chsl.str.cgsl	ASD Severity	1_very	mediated	2 CG sleep	0.006	0.003	0.011	1.000	0.017
asd.sev1.str.cgsl	ASD Severity	1_very	mediated	2 CG sleep	0.079	0.050	0.114	1.000	0.220
b.cgsl[41]	ASD Severity	1_very	direct	2 CG sleep	0.152	-0.003	0.309	0.972	0.420
asd.sev2.chsl.cgsl	ASD Severity	2_extreme	mediated	2 CG sleep	0.118	0.026	0.212	0.994	0.267
asd.sev2.chsl.str.cgsl	ASD Severity	2_extreme	mediated	2 CG sleep	0.006	0.001	0.012	0.994	0.013
asd.sev2.str.cgsl	ASD Severity	2_extreme	mediated	2 CG sleep	0.100	0.061	0.148	1.000	0.227
b.cgsl[42]	ASD Severity	2_extreme	direct	2 CG sleep	0.218	-0.025	0.460	0.961	0.493
asd.symp.chsl.cgsl	ASD Symptoms	AIM	mediated	2 CG sleep	0.088	0.071	0.107	1.000	0.555
asd.symp.chsl.str.cgsl	ASD Symptoms	AIM	mediated	2 CG sleep	0.005	0.003	0.007	1.000	0.028
asd.symp.str.cgsl	ASD Symptoms	AIM	mediated	2 CG sleep	0.049	0.032	0.067	1.000	0.308
b.cgsl[32]	ASD Symptoms	AIM	direct	2 CG sleep	0.017	-0.028	0.063	0.771	0.109
asd.mjev.elop.chsl.cgsl	ASD Major Adversities	Elope	mediated	2 CG sleep	0.086	0.055	0.119	1.000	0.457
asd.mjev.elop.chsl.str.cgsl	ASD Major Adversities	Elope	mediated	2 CG sleep	0.004	0.002	0.007	1.000	0.023
asd.mjev.elop.str.cgsl	ASD Major Adversities	Elope	mediated	2 CG sleep	0.027	0.016	0.041	1.000	0.142
b.cgsl[28]	ASD Major Adversities	Elope	direct	2 CG sleep	0.071	-0.011	0.153	0.956	0.378

asd.mjev.erhp.chsl.cgsl	ASD Major Adversities	ER mental	mediated	2 CG sleep	0.037	-0.045	0.118	0.812	0.281
asd.mjev.erhp.chsl.str.cgsl	ASD Major Adversities	ER mental	mediated	2 CG sleep	0.002	-0.002	0.007	0.812	0.014
asd.mjev.erhp.str.cgsl	ASD Major Adversities	ER mental	mediated	2 CG sleep	0.034	0.012	0.063	0.999	0.259
b.cgsl[30]	ASD Major Adversities	ER mental	direct	2 CG sleep	0.059	-0.150	0.270	0.705	0.446
asd.mjev.otml.chsl.cgsl	ASD Major Adversities	Other comorb	mediated	2 CG sleep	0.046	0.017	0.075	0.999	0.481
asd.mjev.otml.chsl.str.cgsl	ASD Major Adversities	Other comorb	mediated	2 CG sleep	0.002	0.001	0.004	0.999	0.024
asd.mjev.otml.str.cgsl	ASD Major Adversities	Other comorb	mediated	2 CG sleep	0.027	0.016	0.041	1.000	0.287
b.cgsl[27]	ASD Major Adversities	Other comorb	direct	2 CG sleep	0.020	-0.055	0.094	0.699	0.207
b.cgsl[33]	Child sleep	Child sleep	direct	2 CG sleep	0.364	0.328	0.399	1.000	1.000

Abbreviations: *AIM* Autism Impact Measure, *ASD* autism spectrum disorder, *CG* caregiver, *ER* emergency room.
